# The Combination of Uric Acid and Hemoglobin Levels Predicts the Incident Risk of Ischemic Heart Disease More Than Uric Acid Alone in Non-Diabetic Koreans: A Longitudinal Study Using National Health Insurance Data

**DOI:** 10.3390/jpm14010007

**Published:** 2023-12-20

**Authors:** Sung-Bum Lee, Hui-Jeong Lee, Ha Eun Ryu, Byoungjin Park, Dong-Hyuk Jung

**Affiliations:** 1 Department of Family Medicine, Soonchunhyang University Bucheon Hospital, Bucheon 22972, Republic of Korea; sblee@schmc.ac.kr (S.-B.L.); lhj@schmc.ac.kr (H.-J.L.); 2 Department of Family Medicine, Yonsei University College of Medicine, Seoul 03722, Republic of Korea; rahahaha@yuhs.ac

**Keywords:** uric acid, hemoglobin, ischemic heart disease, national health insurance data, Korea

## Abstract

Uric acid has been related to cardiovascular disease (CVD). Recently, slightly elevated hemoglobin (Hb) was also shown to be associated with CVD. We retrospectively investigated the joint effect of uric acid and elevated Hb by comparing normal-range uric acid alone on incident ischemic heart disease (IHD) risk in a large cohort of non-diabetic Korean adults using National Health Insurance data. We assessed 16,786 participants without diabetes (8595 men and 8191 women) using extensive cohort data. High Hb was defined as ≥16.4 g/dL in men and 13.8 g/dL in women (>75th percentile). We analyzed the data using two different methods. First, the participants were divided into quartiles according to uric acid levels. Second, subjects were also divided into quartiles: reference (group 1), high uric acid and normal Hb (group 2), normal uric acid and high Hb (group 3), and normal uric acid and high Hb (group 4). We evaluated hazard ratios (HRs) with 95% confidence intervals (CIs) for IHD using multivariate Cox regression analysis over a 50-month follow-up. During the follow-up, 345 (1.9%) participants developed IHD. In the analysis using both uric acid and Hb, compared with the reference group, the HRs for IHD were 1.37 (95% CI, 1.01–1.86) in the second group, 1.63 (95% CI, 1.21–2.21) in the third group, and 1.86 (95% CI, 1.30–2.67) in the fourth group after adjusting for IHD risk factors. Subsequently, patients with high uric acid are more likely to develop incident IHD than control patients. Moreover, we confirmed the joint effects of high uric acid and high hemoglobin on incident IHD. Awareness of these interactions is essential for clinicians. Risk factor management and screening for IHD are part of the routine management of patients with high uric acid and Hb.

## 1. Introduction

The prevalence of hyperuricemia has been rising in recent years, thereby increasing the burden of disease management. Research in the U.S. has suggested that the prevalence of gout among people above 75 doubled from 2.1% to 4.1% in the 1990s [1]. Gout, a clinical condition that arises after prolonged hyperuricemia, is estimated to affect approximately 5.1 million individuals in the U.S. [2]. Another study in the U.K. revealed that overall hyperuricemia increased from 0.26% to 0.95% between 1970 and 1993 [3]. Additionally, elevated uric acid levels have been identified as contributing factors in both metabolic syndrome and hypertension. These conditions are linked to an increased risk of developing cardiovascular diseases (CVDs) [4,5]. The pathogenesis and causality of the association between uric acid and cardiovascular disease (CVD), however, remain controversial because of the strong collinearity of uric acid with metabolic syndrome [6].

The early detection and modification of hyperuricemia are essential health concerns that require attention before progression to comorbidity, leading to a spectrum of complications [7]. Nevertheless, elevated uric acid levels tend to be neglected, as individuals often have no symptoms before the condition advances to gout.

Several studies have shown that high hemoglobin concentrations could increase the risk of CVDs [8,9]. In other words, thromboembolic complications and CVDs are the most common complications in patients with polycythemia vera (PV) [10]. Thromboembolic events in patients with PV often occur years before the disease is diagnosed; this situation affects 12–15% of patients [11,12]. Nevertheless, the impact of elevated hemoglobin concentrations on CVD prevalence within the general populace continues to be contentious. The effect differs among the subtypes of CVDs [13].

Hyperuricemia is associated with polycythemia. Despite elevated serum urate levels, fractional urate excretion remains low in PV patients [14]. Because renal plasma flow is reduced in polycythemic patients, excretion function decreases, increasing uric acid levels [15]. However, the joint effects of high uric acid and hemoglobin on incident ischemic heart disease (IHD) are unknown. Consequently, our study conducted a retrospective analysis of the combined effects of elevated uric acid and hemoglobin on incident IHD risk in a substantial Korean cohort without diabetes mellitus.

## 2. Methods

### 2.1. Study Population

**Figure 1:**
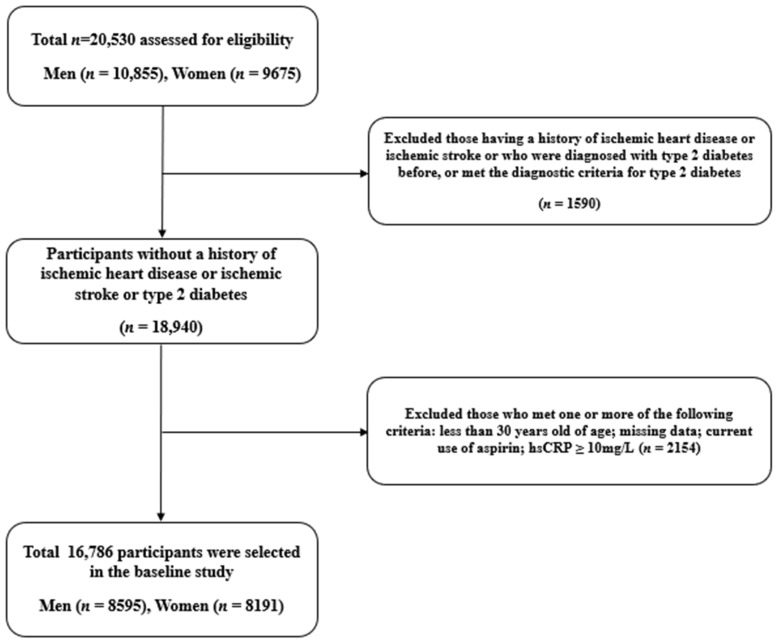
Flow diagram for the study population selection.

This study analyzed data from the HERAS-HIRA database. The cohort consisted of 20,530 subjects who underwent health examinations at the Yonsei University Gangnam Severance Hospital Health Promotion Center. Most subjects lived in the Gangnam area, a metropolitan region in Korea. The 20,530 participants in the baseline survey visited the hospital between 2006 and 2010 while being assessed for about 50 months from the initial enrollment. We excluded participants meeting one or more criteria: missing data; a history of IHD, ischemic stroke, and type 2 diabetes; those under 30 years old; current use of aspirin; high-sensitivity C-reactive protein (hsCRP) ≥ 10 mg/L. Consequently, 16,786 participants without type 2 diabetes, IHD, and ischemic stroke were included in the final analysis (Figure 1).

### 2.2. Measurements and Outcomes

We gleaned a comprehensive questionnaire detailing their medical history and health-related behaviors. Smoking status was classified into three groups: non-smoker, ex-smoker, or current smoker. Alcohol consumption was evaluated, with “regular drinkers” defined as those who ingested over 140 g of alcohol weekly [16]. The anthropometric and laboratory methodology was described in detail in a previous study using the HERAS–HIRA dataset [17]. Hypertension criteria included a systolic blood pressure (SBP) of 140 mmHg or more, a diastolic blood pressure (DBP) of 90 mmHg or more or using anti-hypertensive drugs [18]. The outcome of our study was IHD, which included cases of angina pectoris or acute myocardial infarction (ICD-10 code I20 and I21, respectively) that were identified after participants’ enrolment. For ascertaining initial and post-survey outcomes, we utilized the unique 13-digit identification numbers allocated to every participant by the Korean HIRA until 31 December 2010.

### 2.3. Statistical Analysis

The 16,786 participants were classified into quartiles by their uric acid levels. Furthermore, to assess the joint effect of uric acid and hemoglobin on incident IHD, we divided the study participants into four groups: reference group, high uric acid group, high Hb group, and high uric acid and high Hb group. The high Hb group was defined as Hb levels above 16.3 g/dL and 13.8 g/dL in men and women, respectively (>75th percentile). The high uric acid group was defined as ≥6.5 mg/dL and ≥4.6 mg/dL for men and women, respectively, based on the 75th percentile [19]. We compared baseline characteristics of the study population across different groups using an analysis of variance (ANOVA) model for continuous variables and a chi-squared test for categorical variables. Kaplan–Meier curves assessed the cumulative incidence of IHD, while log-rank tests determined if the distributions of the cumulative incidence of IHD differed between groups. For the predictive power for IHD incidence, pairwise comparisons of receiver operating characteristic (ROC) curves were used to compare uric acid alone versus its combination with hemoglobin. Considering the first group as the reference, we calculated hazard ratios (HRs) and 95% confidence intervals (CIs) for IHD using multivariate Cox proportional hazards regression. All data analysis was conducted with SAS version 9.4 (SAS Institute Inc., Cary, NC, USA) with statistical significance at *p* < 0.05.

## 3. Results

### 3.1. Baseline Characteristics

This study included 16,786 participants in the HERAS-HIRA database, which were used to access first outcomes with an IHD incidence of 345 individuals (219 men and 126 women) and an average 50-month follow-up. Table 1 shows the biochemical and demographic characteristics of the 16,786 men and women according to the uric acid level quartile. The overall study population’s average age and body mass index were 46.2 ± 9.6 years and 23.4 ± 3.0 kg/m^2^, respectively. The mean body mass index, blood pressure, fasting plasma glucose, total cholesterol, triglyceride, hsCRP, and the proportion of alcohol intake, hypertension, and chronic kidney disease increased as uric acid levels increased. To assess the joint effect of uric acid and hemoglobin concentrations on incident IHD, we subdivided the study participants into four groups: reference (group 1); high uric acid and normal hemoglobin (group 2); normal uric acid and high hemoglobin (group 3); and normal uric acid and high hemoglobin (group 4). Participants with high uric acid and hemoglobin levels were similar in their mean age to the reference group but had higher blood pressure, fasting plasma glucose, total cholesterol, and hsCRP. The proportions of current smokers, alcohol intake, hypertension, and chronic kidney disease were highest in the group with both high uric acid and hemoglobin (Table 2).

### 3.2. Uric Acid Alone vs. the Joint Effect of Uric Acid and Hemoglobin

**Figure 2:**
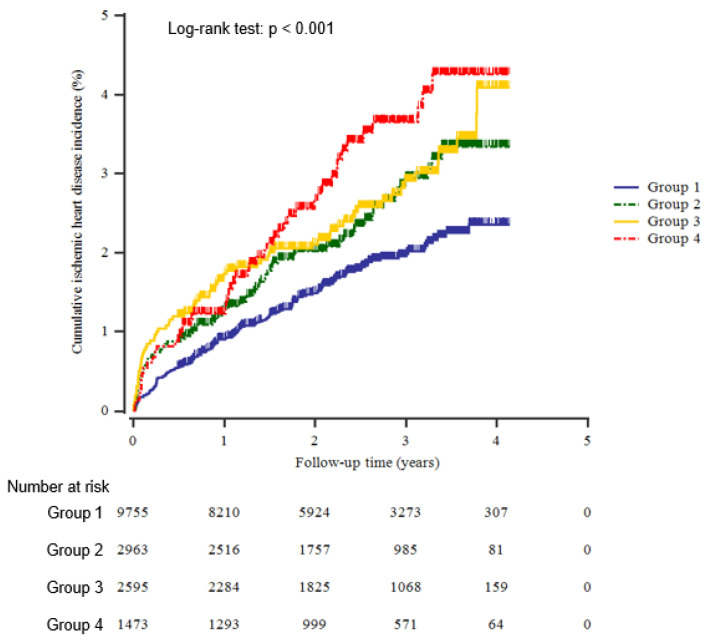
Kaplan–Meier plots for cumulative new-onset IHD after the baseline survey.

Table 3 provided the multivariate Cox proportional hazards regression analysis results for predicting incident IHD based on the uric acid quartiles. The HRs of incident IHD were 1.53 (95% CI, 1.14–2.03) in the fourth quartile of uric acid after adjusting for age and sex. These positive associations were similarly observed after additional adjusting for lifestyle factors and major metabolic parameters (models 2 and 3). In Table 4, which was used to assess the joint effect of uric acid and hemoglobin, the group with both risk factors significantly increased by 97%, 89%, and 86% in models 1, 2, and 3 compared with the reference group (*p* < 0.001, respectively). The cumulative IHD for each group, subdivided by the control or elevation of uric acid and hemoglobin levels, is illustrated in Figure 2. The incidence of IHD was highest in group 4 (elevated uric acid with elevated hemoglobin), followed by group 3 (control of uric acid with elevated hemoglobin). Group 1 (control of uric acid and hemoglobin) had the lowest incidence of IHD, and group 2 (elevated uric acid with control of hemoglobin) had a higher incidence than group 1; however, it had a lower incidence of IHD than group 3 and group 4. A sex-based subgroup analysis showed that the HRs for incident IHD tended to be slightly higher in women than in men (Table 5).

Lastly, we compared the ROC curves to analyze the two methods’ power to predict the stratification of IHD. A comparison of using two risk factors simultaneously with using only one risk factor, uric acid, showed that the former was superior to the latter in predicting new-onset IHD (*p* = 0.029) (Table 6). The AUC, sensitivity, and specificity were 0.568, 53.6%, and 58.4% for uric acid with hemoglobin and 0.535, 30.7%, and 76.7% for uric acid alone, respectively. The cutoff value was greater than controls according to uric acid levels and hemoglobin, greater than the third quartile according to the uric acid level quartiles, and 5.0 mg/dL for uric acid levels. Additionally, the negative predictive value for group 1 was 98.5%, and the positive predictive value for group 4 was 2.4%.

## 4. Discussion

High uric acid levels with elevated hemoglobin levels could predict new-onset IHD in non-diabetic participants by using extensive cohort data. Serum uric acid could be an independent predictor of IHD irrespective of age, sex, body mass index, smoking status, alcohol intake, physical activity, blood pressure, fasting plasma glucose, HDL cholesterol, hsCRP, and chronic kidney disease, especially in participants with high hemoglobin levels.

The relationship between uric acid and cardiovascular disease is widely acknowledged. Several studies have explored this connection [5,20]. Nevertheless, it remains unclear whether uric acid serves as a risk indicator for cardiovascular disease or if management to reduce uric acid influences the incidence of cardiovascular disease. While uric acid is independently correlated with cardiovascular risk [21], one study showed that this association was due to uric acid’s strong collinearity with traditional risk factors such as metabolic syndrome [6]. Other research has indicated that the pathophysiology and results of the link between uric acid and cardiovascular disease are still disputable [22]. However, our study revealed that groups with high levels of uric acid were positively associated with an increased incidence of IHD, and it also suggested that groups with high hemoglobin levels have an increased incidence of IHD. Above all, this is a distinguished study that analyzed the combined effect of uric acid and hemoglobin on the prevalence of IHD by comparing groups with these factors to those within control levels.

We found a correlation between increased levels of uric acid and hemoglobin and IHD after four years of following many subjects. The relationship remained significant even after adjusting for essential determinants of metabolic syndrome, such as blood pressure, fasting plasma glucose, and HDL cholesterol. Notably, we found a joint effect of uric acid and Hb. The effect persisted throughout the follow-up period.

Although it is demanding to reveal the mechanism of the joint effect of uric acid and hemoglobin on IHD, presumed mechanisms can be comprehended by understanding the pathogenesis of elevated uric acid and IHD and the pathogenesis of increased hemoglobin and IHD, respectively.

Uric acid could indicate that incidences of metabolic syndrome and cardiovascular disease are linked to oxidative stress despite being a major antioxidant in human plasma [23]. Uric acid might serve as an antioxidant in plasma or a pro-oxidant within the cell [24]. The hydrophobic environment formed by lipids negatively affects the antioxidant properties of uric acid. When lipids are oxidized, they can transform uric acid into an oxidant. Uric acid is the product of purine metabolism catalyzed by xanthine oxidase. In metabolism, reactive oxygen, including superoxide, is simultaneously generated along with uric acid. The superoxide anion, concomitant with uric acid production, interacts with nitric oxide, a well-known vasodilator [25]. In addition, several studies have indicated that uric acid intake into cells is associated with a reduction in the bioavailability of nitric oxide (NO), which can lead to endothelial dysfunction [25,26]. In other words, elevated uric acid can cause arteriolar diseases such as hypertension and cardiovascular disease [27,28].

Blood viscosity is largely contingent upon the quantity of red blood cells. Higher hematocrit levels, defined as the ratio of the volume of red blood cells to the total blood volume, can markedly intensify the blood’s viscosity. It decreases the blood flow rate and increases peripheral resistance, thereby reducing the oxygen supply to various organs such as the heart and brain [29]. Moreover, elevated hematocrit concentrations amplify platelet activation and oxidative stress by releasing ADP in response to iron accumulation, which can induce a significantly increased risk of CVDs [30,31].

Despite the large amount of data, our study had several limitations. First, this research did not reflect the effect of changes in uric acid and hemoglobin levels, which could influence the incidence of IHD during the follow-up. Second, no data are available on the history of uric-acid-lowering medication, which could impact uric acid levels. Third, participants who were at risk of dying during the follow-up period were excluded because they were considered follow-up loss. A history of respiratory diseases could not be reflected because the data were unavailable. Finally, the study population did not precisely represent the overall Korean population. Subjects participating in health check-up programs are usually interested in health problems and conducted programs in a single health check-up center, causing selection bias.

## 5. Conclusions

In summary, uric acid levels are positively associated with IHD. Elevated hemoglobin is also related to the incidence of IHD. Hemoglobin is more strongly associated with incident IHD than uric acid. In addition, the combined effects of high uric acid and high hemoglobin on the prevalence of IHD were found. Regular checks of uric acid and hemoglobin levels are necessary to discover the development of IHD. Further studies are needed to identify the direct mechanism of the combined effect of uric acid and hemoglobin on IHD.

## Figures and Tables

**Table 1:** Baseline characteristics of the study population according to serum uric acid quartiles.

	Quartile of Serum Uric Acid				*p* Value ^a^	Post Hoc ^b^
	Q1 *n* = 4502	Q2 *n* = 4304	Q3 *n* = 4044	Q4 *n* = 3936		
Age (years)	46.5 ± 9.5	45.9 ± 9.3	45.9 ± 9.5	46.6 ± 9.9	<0.001	a,b,e,f
Male sex (%)	50.8	52.3	47.9	53.9	<0.001	-
Body mass index (kg/m^2^)	22.6 ± 2.8	23.1 ± 2.8	23.5 ± 3.0	24.5 ± 3.1	<0.001	a,b,c,d,e,f
SBP (mmHg)	120.0 ± 15.1	121.3 ± 15.2	122.0 ± 15.5	125.3 ± 15.7	<0.001	a,b,c,e,f
DBP (mmHg)	74.8 ± 9.8	75.7 ± 10.0	76.3 ± 10.0	78.5 ± 10.1	<0.001	a,b,c,e,f
Hemoglobin (g/dL)	14.1 ± 1.7	14.4 ± 1.7	14.4 ± 1.6	14.8 ± 1.6	<0.001	a,b,c,e,f
Fasting plasma glucose (mg/dL)	90.4 ± 9.3	90.9 ± 9.2	91.5 ± 9.9	93.3 ± 10.5	<0.001	b,c,d,e,f
Total cholesterol (mg/dL)	183.0 ± 31.2	188.1 ± 33.0	192.1 ± 33.0	199.3 ± 34.7	<0.001	a,b,c,d,e,f
Triglyceride (mg/dL)	107.3 ± 67.0	117.2 ± 78.9	125.9 ± 92.6	152.7 ± 100.4	<0.001	a,b,c,d,e,f
HDL cholesterol (mg/dL)	55.0 ± 12.5	53.6 ± 12.5	53.1 ± 12.9	50.9 ± 12.2	<0.001	a,b,c,e,f
hsCRP (mg/L)	0.8 ± 1.2	1.0 ± 1.3	1.0 ± 1.3	1.4 ± 1.5	<0.001	a,b,c,e,f
Current smoker (%)	22.4	24.2	23.7	28.2	<0.001	-
Alcohol intake (%)	41.2	42.8	43.5	45.5	0.001	-
Regular exercise (%)	32.3	31.5	30.3	30.5	0.182	-
Hypertension (%)	15.7	19.0	20.5	29.6	<0.001	-
Chronic kidney disease (%)	0.7	1.1	2.0	4.8	<0.001	-

^a^ *p* values were calculated using 1-way ANOVA or Pearson’s chi-square test. ^b^ Post hoc analysis with the Bonferroni method: a, Q1 versus Q2; b Q1 versus Q3; c, Q1 versus Q4; d, Q2 versus Q3; e, Q2 versus Q4; and f, Q3 versus Q4.

**Table 2:** Baseline characteristics according to uric acid levels and hemoglobin.

	Control of Hemoglobin		High Hemoglobin		*p* Value ^a^	Post Hoc ^b^
	Control of Uric Acid (*n* = 9755)	High Uric Acid (*n* = 2963)	Control of Uric Acid (*n* = 2595)	High Uric Acid (*n* = 1473)		
Age (years)	46.1 ± 9.5	46.6 ± 10.0	45.9 ± 9.2	46.6 ± 9.8	0.021	-
Male sex (%)	50.8	54.5	50.1	49.1	<0.001	-
Body mass index (kg/m^2^)	22.9 ± 2.8	24.2 ± 3.1	23.4 ± 3.0	24.6 ± 3.1	<0.001	a,b,c,d,e,f
SBP (mmHg)	120.1 ± 15.1	124.0 ± 15.5	124.1 ± 15.4	126.8 ± 16.1	<0.001	a,b,c,e,f
DBP (mmHg)	74.9 ± 9.8	77.6 ± 10.0	78.0 ± 10.0	79.7 ± 10.2	<0.001	a,b,c,e,f
Fasting plasma glucose (mg/dL)	90.9 ± 9.3	93.0 ± 10.2	91.0 ± 10.1	93.4 ± 11.0	<0.001	a,c,d,f
Total cholesterol (mg/dL)	185.2 ± 31.9	196.3 ± 34.0	194.9 ± 33.6	204.0 ± 35.2	<0.001	a,b,c,e,f
Triglyceride (mg/dL)	112.1 ± 73.3	145.4 ± 98.5	131.0 ± 100.7	158.4 ± 97.5	<0.001	a,b,c,d,e,f
HDL cholesterol (mg/dL)	54.0 ± 12.6	51.3 ± 12.3	53.9 ± 12.9	50.8 ± 12.1	<0.001	a,c,d,f
C-reactive protein (mg/L)	0.9 ± 1.3	1.3 ± 1.6	1.0 ± 1.3	1.3 ± 1.4	<0.001	a,b,c,d,f
Current smoker (%)	21.6	25.6	30.0	32.4	<0.001	-
Alcohol intake (%)	41.9	45.1	44.7	45.3	0.001	-
Regular exercise (%)	31.5	31.4	30.9	29.3	0.417	-
Hypertension (%)	16.7	27.3	23.7	31.4	<0.001	-
Chronic kidney disease (%)	1.1	4.2	1.7	5.2	<0.001	-

^a^ *p* values were calculated using 1-way ANOVA or Pearson’s chi-square test. ^b^ Post hoc analysis with the Bonferroni method: a, Q1 versus Q2; b, Q1 versus Q3; c, Q1 versus Q4; d, Q2 versus Q3; e, Q2 versus Q4; and f, Q3 versus Q4.

**Table 3:** Hazard ratios and 95% confidence intervals for new-onset ischemic heart disease according to uric acid quartiles.

		Quartile of Serum Uric Acid			
		Q1	Q2	Q3	Q4
New cases of ischemic heart disease, n		84	82	73	106
Mean follow-up, years		2.4 ± 1.1	2.4 ± 1.1	2.3 ± 1.1	2.4 ± 1.1
Pearson-years of follow-up		10,866	10,224	9452	9273
Incidence rate/1000 person-years		7.7	8.0	7.7	11.4
Model 1	HR (95% CI)	1.00	1.12 (0.83–1.52)	1.09 (0.80–1.49)	1.53 (1.14–2.03)
	*p* value	–	0.471	0.588	0.004
Model 2	HR (95% CI)	1.00	1.09 (0.79–1.50)	1.07 (0.77–1.49)	1.45 (1.06–1.98)
	*p* value	–	0.614	0.692	0.020
Model 3	HR (95% CI)	1.00	1.09 (0.79–1.50)	1.05 (0.75–1.47)	1.41 (1.03–1.94)
	*p* value	–	0.617	0.767	0.034

Model 1: adjusted for age and sex. Model 2: adjusted for age, sex, body mass index, smoking status, alcohol intake, and physical activity. Model 3: adjusted for age, sex, body mass index, smoking status, alcohol intake, physical activity, mean arterial blood pressure, fasting plasma glucose, HDL cholesterol, high sensitivity C-reactive protein level, and chronic kidney disease.

**Table 4:** Hazard ratios and 95% confidence intervals for new-onset ischemic heart diseases according to uric acid levels and hemoglobin.

		Control of Hemoglobin		High Hemoglobin	
		Control of Uric Acid	High Uric Acid	Control of Uric Acid	High Uric Acid
New cases of ischemic heart disease, n		160	68	70	47
Mean follow-up, years		2.3 ± 1.1	2.3 ± 1.1	2.6 ± 1.1	2.5 ± 1.0
Pearson-years of follow-up		22,742	6784	6646	3643
Incidence rate/1000 people-years		7.0	10.0	10.5	12.9
Model 1	HR (95% CI)	1.00	1.39 (1.05–1.85)	1.67 (1.26–2.21)	1.97 (1.42–2.74)
	*p* value	–	0.022	<0.001	<0.001
Model 2	HR (95% CI)	1.00	1.38 (1.02–1.86)	1.60 (1.19–2.17)	1.89 (1.32–2.70)
	*p* value	–	0.036	0.002	<0.001
Model 3	HR (95% CI)	1.00	1.37 (1.01–1.86)	1.63 (1.21–2.21)	1.86 (1.30–2.67)
	*p* value	–	0.043	0.001	<0.001

Model 1: adjusted for age and sex. Model 2: adjusted for age, sex, body mass index, smoking status, alcohol intake, and physical activity. Model 3: adjusted for age, sex, body mass index, smoking status, alcohol intake, physical activity, mean arterial blood pressure, fasting plasma glucose, HDL cholesterol, high-sensitivity C-reactive protein level, and chronic kidney disease.

**Table 5:** Hazard ratios and 95% confidence intervals for new-onset ischemic heart disease according to uric acid levels and hemoglobin by sex.

		Control of Hemoglobin		High Hemoglobin	
		Control of Uric Acid	High Uric Acid	Control of Uric Acid	High Uric Acid
Model 1	Men	1.00	1.33 (0.93–1.90)	1.60 (1.12–2.29)	1.91 (1.22–2.98)
	Women	1.00	1.45 (0.90–2.35)	1.74 (1.09–2.77)	1.94 (1.17–3.20)
Model 2	Men	1.00	1.27 (0.88–1.83)	1.49 (1.02–2.16)	1.70 (1.06–2.73)
	Women	1.00	1.56 (0.91–2.66)	1.83 (1.09–3.07)	2.12 (1.21–3.73)
Model 3	Men	1.00	1.22 (0.84–1.77)	1.50 (1.03–2.19)	1.64 (1.01–2.64)
	Women	1.00	1.62 (0.95–2.78)	1.89 (1.12–3.18)	2.24 (1.26–3.97)

The same modeling applies except for sex, as shown in Table 4.

**Table 6:** Uric acid quartiles versus the combination of uric acid levels with hemoglobin for predicting ischemic heart disease.

	Pairwise Comparison of AUC			Classifying Ability for Ischemic Heart Disease				
	Difference	95% CI	*p* Value	Cutoff Value	Sensitivity (%)	Specificity (%)	AUC	*p* Value
Uric acid with hemoglobin vs. uric acid quartiles	0.032	0.003 to 0.062	0.029					
Uric acid with hemoglobin				>Controls	53.6	58.4	0.568	<0.001
Uric-acid-level quartiles				>3rd quartile	30.7	76.7	0.535	0.024

AUC area under the receiver operating characteristic curve.

## Data Availability

The datasets used and analyzed in the current study are available from the corresponding author upon reasonable request.
